# A serologic marker attenuated live vaccine protects piglets against highly pathogenic porcine reproductive and respiratory syndrome virus infection

**DOI:** 10.1186/s13567-025-01526-8

**Published:** 2025-04-24

**Authors:** Jiakai Zhao, Hong Duan, Xu Chen, Binbin Ren, Qianyi Zhu, Pinpin Ji, Yueting Chang, Yani Sun, Qin Zhao

**Affiliations:** 1 Department of Preventive Veterinary Medicine, College of Veterinary Medicine, Northwest A&F University; Yangling Observing and Experimental Station of National Data Center of Animal Health, Ministry of Agriculture, Yangling, 712100 China; 2 College of Veterinary Medicine, Henan Agricultural University, Zhengzhou, 450046 Henan China

**Keywords:** Serologic marker or differentiation of infected and vaccinated animal (DIVA) vaccines, porcine reproductive and respiratory syndrome virus (PRRSV), infectious cDNA clone, HP-PRRSV, nucleocapsid (N) protein

## Abstract

Currently, there are no commercial serologic marker or differentiation of infected and vaccinated animal (DIVA) vaccines for the eradication of porcine reproductive and respiratory syndrome virus (PRRSV) infection from pig farms. In a previous study, a nanobody-based competitive ELISA (cELISA) was specifically developed to detect anti-genotype 2 PRRSV (PRRSV-2) antibodies. On the basis of the epitope recognized by the nanobody and the prevalence of PRRSV-2 infection in China, a DIVA vaccine candidate strain was designed and evaluated in the present study. First, an infectious cDNA clone based on the genomic sequence of the highly pathogenic PRRSV-2 (HP-PRRSV) isolate SX-HD was constructed and named rSX-HD. Using the infectious clone as the backbone, a chimeric infectious cDNA clone in which the gene encoding the nucleocapsid (N) protein was replaced with the gene encoding the genotype 1 PRRSV N protein was generated and named rSX-HD^2M1^. The chimeric PRRSV rSX-HD^2M1^ was subsequently rescued successfully in Marc-145 cells, which were then passaged for 120 generations for attenuation. A safety study indicated that rSX-HD^2M1^-F120 is not pathogenic to piglets. In vivo inoculation and challenge experiments suggested that rSX-HD^2M1^-F120 vaccination significantly reduced serum viral loads and lung tissue lesions and that vaccinated piglets did not show any clinical symptoms or histopathological changes. Furthermore, this recombinant marker virus, in conjunction with the previously developed nanobody-based cELISA, enables serological differentiation between marker virus-infected animals and those infected with wild-type PRRSV-2. These results suggest that rSX-HD^2M1^-F120 is a good candidate for providing a live attenuated DIVA vaccine against PRRSV-2 infection in piglets.

## Introduction

Porcine reproductive and respiratory syndrome virus (PRRSV), the causative agent of PRRS, is an enveloped, nonsegmented, positive-strand RNA virus within the family *Arteriviridae* and genus *Betaarterivirus* [1]. PRRSV infection causes reproductive disorders in pregnant sows and respiratory complications in pigs of all ages [2]. PRRSV can be further divided into two main types on the basis of genetic, antigenic and pathogenic differences: *Betaarterivirus suid 1* (PRRSV-1) and *Betaarterivirus suid 2* (PRRSV-2) according to the ICTV in 2021. PRRSV-1 is prevalent mainly in Europe, and PRRSV-2 is prevalent in America and Asia. However, in recent years, several subtypes of the two genotypes of PRRSV have been found across North America, Europe, and Asia [3, 4].

Since the disease was first identified in North America in the late 1980s, PRRS has resulted in considerable economic losses [5]. Furthermore, highly pathogenic PRRSV-2 (HP-PRRSV), characterized by hyperpyrexia, high morbidity and mortality, occurred in China in 2006 and caused many deaths in piglets [6]. Recently, PRRSV-2 NADC30-like and NADC34-like strains, which have amino acid (aa) deletions corresponding to the Nsp2 gene of VR-2332, have been frequently reported in China [7, 8]. In Europe, attenuated PRRSV-1 strain vaccines have been used for the prevention and control of PRRSV, whereas in China and the United States, attenuated PRRSV-2 strain vaccines have been used [9]. Currently, attenuated PRRSV-2 vaccines, including Ingelvac PRRS MLV/RespPRRS MLV, HuN4-F112, JXA1-P80, TJM-F92 and GDr180, are universally used in China and do not provide completely effective protection against heterologous PRRSV strains [10, 11]. Additionally, another major limitation of the current live-attenuated PRRSV vaccines is that no commercial marker vaccine (distinguishing between natural infection and vaccine immunity) and a matching serological differential diagnosis are available, which makes eradicating the disease from pig farms difficult [12].

The genome of PRRSV is approximately 15 kb in length and contains at least ten open reading frames (ORFs) [13]. Two polyproteins, PP1a and PP1ab, are encoded by ORF1a and ORF1b, respectively, and they constitute more than two-thirds of the genome at the 5′ terminus and are processed into 16 nonstructural proteins (Nsps): Nsp1α, Nsp1β, Nsp2, Nsp2TF, Nsp2N, Nsp3-6, Nsp7α, Nsp7β, and Nsp8-12 [14]. ORF2-7 encode structural proteins, including five minor envelope proteins (GP2a, E, GP3, GP4, and ORF5a), two major envelope proteins (GP5 and M), and a nucleocapsid protein (N) [15]. As the conserved and most abundant protein of viral particles, the PRRSV-N protein interacts with genomic RNA to form the viral nucleocapsid, participates in virion assembly, and is an ideal target protein for the diagnosis and labelling of vaccines [16].

Compared with the corresponding wild-type viruses, differentiated infected from vaccinated animal (DIVA) vaccine candidates should lack at least one antigenic component (the so-called serologic marker antigen) [17]. Therefore, only wild-type virus-infected rather than vaccinated animals can produce antibodies against the marker antigen. Consequently, serological assays detecting specific antibodies against the marker antigen can be used to identify wild-type virus-infected animals in the vaccinated population [12]. The use of DIVA vaccines is preferred or even mandatory in animal health campaigns aimed at controlling and eradicating important animal diseases [17]. Typically, live-attenuated DIVA vaccines are generated through the deletion of an entire gene encoding an immunogenic, nonessential protein [17]. While it is technically straightforward in the case of some double-stranded DNA viruses, such as *Pseudorabies virus* (Suid herpesvirus 1), it is very difficult to delete an entire gene of a smaller RNA genome virus, such as PRRSV, whose genes are all essential for productive viral infection [18, 19]. An alternative approach to developing live-attenuated DIVA vaccines for RNA viruses is to selectively eliminate a small protein fragment or an epitope instead of deleting the whole protein [20–22]. Previously, we developed a competitive ELISA (cELISA) based on nanobodies to specifically detect anti-PRRSV-2 antibodies in pig serum and verified the antigen epitope of the nanobody [23]. Given the absence of this epitope in PRRSV-1 and the predominant PRRSV-2 infection in China, a chimeric PRRSV strain as a marker vaccine candidate was designed to replace the PRRSV-2 N protein on the basis of the HP-PRRSV gene backbone with that of PRRSV-1 via reverse genetics technology. Therefore, candidate strain immunization and natural PRRSV-2 infection can be distinguished via nanobody-based cELISAs.

Therefore, in this study, recombinant and chimeric PRRSV viruses were constructed via infectious clone technology, after which the chimeric virus was passaged for 120 passages in vitro to obtain an attenuated strain. Animal experiments confirmed that the strain had a good protective effect against HP-PRRSV infection. More importantly, the combination of vaccines with cELISA can distinguish between natural PRRSV-2 infection and marker vaccine candidate immunization. Our study provides a novel strategy for the development of a PRRSV-2 marker vaccine and subsequent eradication of PRRSV-2-infected pigs on pig farms.

## Materials and methods

### Plasmid, virus, vaccines and cells

The pBAC vectors were used as the backbone for constructing PRRSV infectious clones and were characterized in a previous study [24]. The PRRSV-2 SX-HD strain was isolated and identified from a field case of PRRS in Shaanxi Province, China. The commercial attenuated live vaccine TJM-F92 (HP-PRRSV) was purchased from Sinovet Biopharmaceutical Co., Ltd. (Jiangsu). Marc-145 cells (ATCC, USA) were cultured in Dulbecco’s minimal Eagle’s medium (DMEM; Life Technologies Corp., Grand Island, NY, USA) supplemented with 10% fetal bovine serum (FBS; Gibco; Thermo Fisher Scientific, Inc.) and penicillin/streptomycin in a humidified 37 °C, 5% CO_2_ incubator. The cells were used for PRRSV propagation and titration. The monoclonal antibody (mAb) 6D10 against the PRRSV-1 and -2 N proteins and the nanobody Nb1 specifically against the PRRSV-2 N protein were maintained in our laboratory [23, 25].

### Construction of the recombinant PRRSV-2 cDNA clone

**Figure 1:**
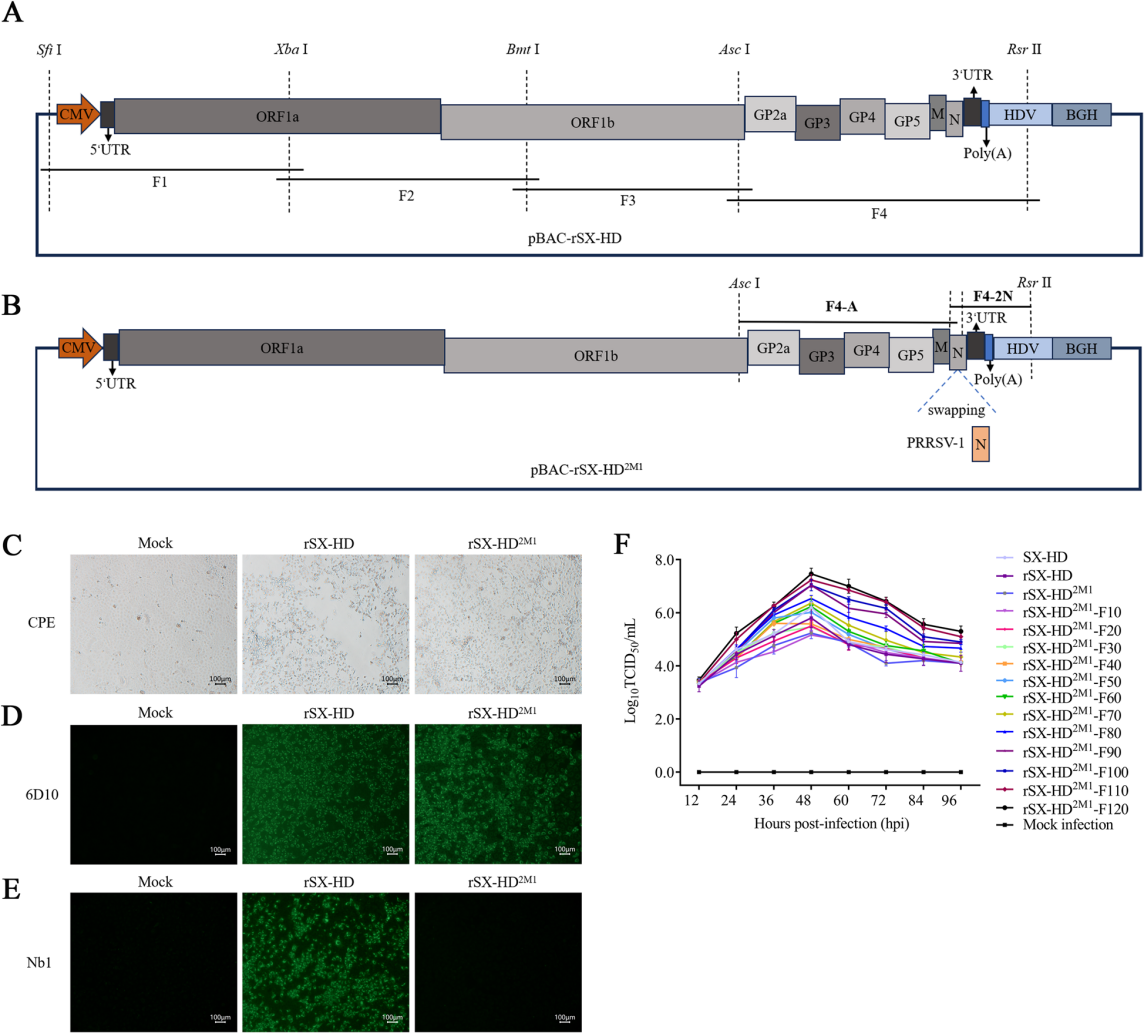
**Construction and rescue of the recombinant PRRSV rSX-HD and rSX-HD**^**2M1**^ **strains**. **A** Schematic diagram of the PRRSV full-length infectious clone pBAC-rSX-HD. **B** Schematic diagram of the chimeric PRRSV full-length infectious clone pBAC-rSX-HD^2M1^. **C** The two recombinant PRRSV strains were successfully rescued, as confirmed by the cytopathic effect (CPE) in Marc-145 cells. **D** Identification of the rescued PRRSV rSX-HD and rSX-HD^2M1^ strains via IFA via the mAb 6D10 in Marc-145 cells. **E** Identification of the rescued PRRSV rSX-HD and rSX-HD^2M1^ strains via IFA of Marc-145 cells with Nb1. **F** Growth kinetics of the parental SX-HD, recombinant rSX-HD and rSX-HD^2M1^ strains and different generations of rSX-HD^2M1^.

The full-length cDNA infectious clone of the PRRSV-2 SX-HD isolate was designed (Figure 1A) and constructed on the basis of a previous study with some modifications [24]. Briefly, four primer pairs were designed to amplify the complete genome of the PRRSV-2 SX-HD strain. Total RNA was extracted from the PRRSV-2 SX-HD stock and reverse transcribed into four distinct overlapping regions via the PrimeScript 1st Strand cDNA Synthesis Kit (TaKaRa, Dalian, China). Each amplicon was inserted into the pEASY™-Blunt simple cloning vector (TransGen Biotech, Beijing, China). After sequencing, the four fragments were introduced into the pBAC-SD16 ^*FL*^-AM vector in turn [24]. The full-length cDNA infectious clone was subsequently constructed and named pBAC-rSX-HD.

Previously, we developed a nanobody-based cELISA for specifically detecting anti-PRRSV-2 antibodies in pig serum. The candidate strain of the PRRSV-2 marker vaccine was subsequently designed and constructed on the basis of the identification of the epitope recognized by the nanobody and the prevalence of PRRSV-2 infection in China (Figure 1B). Briefly, the genes encoding the PRRSV-2 N protein in pBAC-rSX-HD were replaced with the genes encoding the PRRSV-1 N protein from the PRRSV-1 GZ11-G1(KF001144) isolate [26]. After chimeric PRRSV is used to immunize pigs, nanobody-based cELISA can detect antibodies against the wild-type PRRSV-2 isolate but not the candidate strain (chimeric PRRSV). The PRRSV-1 GZ11-G1 strain was used as a template for the amplification of genes encoding the N protein with the primers *Asc* I-F, GZ11-N-R, *Rsr* II-R, and GZ11-N-F (Table 1). The two fragments were named F4-A and F4-2 N, respectively. The recovered products of F4-A and F4-2 N were fused by overlapping fusion PCR. The obtained products were then directly double-digested with *Asc* I and *Rsr* II at 37 °C and ligated into the pBAC-rSX-HD plasmid, which was also double-digested with the same restriction enzymes. After overnight ligation at 16 °C via T4 DNA ligase (NEB), the recombinant construct was transformed into DH10B competent cells (TransGen Biotech). Finally, the full-length cDNA clone pBAC-rSX-HD^2M1^ was confirmed by sequencing and obtained for the following experiments (Figure 1B).

**Table 1:** **Primers used for amplification of the sequences of F4-A and F4-2 N**

Primers	Sequences (5′–3′)
*Asc* I-F	GGCGCGCC AGAAAGGGAAAAT
GZ11-N1-R	CTCTGATTTTTACCGGCCATACTTGACGAG
GZ11-N1-F	ATGGCCGGTAAAAATCAGAG
*Rsr* II-R	CGGACCG CGAGGAGGTGGAGAT

Restriction sites are underlined.

### Rescue of wild-type and chimeric PRRSV

To rescue wild-type rSX-HD and chimeric rSX-HD^2M1^, Marc-145 cells were cultured in 6-well plates and reached approximately 80% confluence. Then, the pBAC-rSX-HD and pBAC-rSX-HD^2M1^ plasmids were separately transfected into the cells via X-tremeGENE HP DNA Transfection Reagent according to the manufacturer’s instructions (Roche, Basel, Switzerland). On day 3 post-transfection, the cells and supernatants were collected and freeze-thawed three times. The supernatants were filtered and then used to infect Marc-145 cells to propagate the rescued virus. The complete genomic sequences of the rescued viruses were confirmed by sequencing and indirect immunofluorescence assay (IFA) using the mAb 6D10 against the PRRSV-1 and -2 N proteins and Nb1 specifically against the PRRSV-2-N protein [23]. The two rescued viruses were separately named PRRSV rSX-HD and rSX-HD^2M1^.

### PRRSV attenuation

To attenuate chimeric PRRSV, traditional methods of serial passage in Marc-145 cells were used in this study [27, 28]. Briefly, rescued rSX-HD^2M1^ cells were inoculated into Marc-145 cells maintained with 3% FBS at 37 °C in a humidified 5% CO_2_ incubator and monitored daily for cytopathic effects (CPEs). When a CPE was observed in 80% of the inoculated cells, the cells were collected and freeze-thawed three times. After centrifugation at 10 000 × *g* for 10 min at 4 °C, the supernatants were collected to inoculate Marc-145 cells again, which were stored at −80 °C. The rescued rSX-HD^2M1^ Marc-145 cells were passaged for 120 generations (F1, F2, F3··········F120). For the different generations, the titres were determined every 10 generations with the tissue culture infective dose 50 (TCID_50_). Briefly, the different generations were serially diluted tenfold in DMEM and added to Marc-145 cells (100 μL per well) in 8 wells. Then, the cells were incubated for 7 days at 37 °C in a humidified incubator under 5% CO_2,_ and the TCID_50_/mL was calculated according to the Reed–Muench method.

### Characterization of viral growth properties

Marc-145 cells were infected with the parental SX-HD virus and different generations of rSX-HD^2M1^ (F1, F10, F20, F30········F120) at the same multiplicity of infection (MOI) of 0.1. After 1 h of incubation at 37 °C, the supernatant was discarded, and the cells were washed with PBS three times. The cell cultures were subsequently supplemented with maintenance medium containing 3% FBS. The viruses were cultured until 96 h post-inoculation (hpi), and the supernatant was collected at 12, 24, 36, 48, 60, 72, 84 and 96 hpi for titration in Marc-145 cells via the endpoint dilution assay. The TCID_50_/mL was recorded and calculated according to the Reed‒Muench method.

### Next-generation sequencing (NGS)

To determine the mutation of different generations, each 10th generation was used to perform next-generation sequencing (NGS). Briefly, total RNA was extracted from different passages of rSX-HD^2M1^ (F1, F10, F20, F30········F120) via the TRIzol method according to the manufacturer’s instructions. The total RNA from the different generations was subsequently separately sent to Shanghai Tanpu Biotechnology Co., Ltd. (Shanghai, China) for next-generation sequencing (NGS). NGS was performed as previously described with some modifications [29]. Briefly, Illumina sequencing technology was used to complete the genome amplification of the virus. The first step was DNase treatment and cleanup. The second strand was subsequently synthesized before library preparation via NextEra XT reagents and sequenced on the NovaSeq 6000 platform (Illumina). Skewer was used to perform read quality trimming, with an additional trimming filter for unreliable sequences after a user-specified quality score was obtained. We used SPAdes and MEGAHIT software to assemble the reads obtained de novo after the removal of the abovementioned contamination sequence.

After the complete genome sequences of different generation strains were assembled, multiple sequence alignments were carried out via the MegAlign program 7.1.0 of the Lasergene software package (DNASTAR, Madison, WI, USA).

### Safety evaluation of the vaccine candidate strain

Twenty 4-week-old PRRSV-free piglets were obtained from a farm in Shaanxi Province and randomly divided into four groups (5 piglets in each group). All the piglets were negative for PRRSV RNA and antibodies against PRRSV. The piglets in groups 1–3 were inoculated intramuscularly with 10^5.0^ TCID_50_ of SX-HD, rSX-HD^2M1^-F1 or rSX-HD^2M1^-F120, respectively. Group 4 was inoculated with DMEM as a mock control. The animals were kept in individual biosafety rooms. Clinical scores were evaluated via a 0–20 scale [30], and rectal temperatures were recorded daily prior to feeding. Blood samples were periodically collected from each piglet and tested for viremia. In addition, nasal and anal swab samples were collected at 1, 3, 5, 7, 10, 14, and 21 days post-challenge (dpc). All the animals were euthanized by intravenous injection of 25 mg/kg body weight sodium pentobarbital at 21 dpc.

### Immunoprotection evaluation of the vaccine candidate strains

Another 20 6-week-old piglets were obtained from a farm in Shaanxi and were randomly divided into four groups. All the piglets were negative for PRRSV RNA and antibodies against PRRSV. Each piglet in group 1 was inoculated intramuscularly with 10^5.0^ TCID_50_ of PRRSV rSX-HD^2M1^-F120. Group 2 was inoculated intramuscularly with 10^5.0^ TCID_50_ TJM-F92 (a commercial attenuated PRRSV vaccine strain). The piglets in groups 3 and 4 were inoculated with DMEM as a control group. The animals were kept in separate rooms. Clinical signs and rectal temperatures were recorded daily prior to feeding. At 21 days post-vaccination (dpv), the HP-PRRSV JXA1 strain (2 mL, 10^5^ TCID_50_/pig) was injected into the neck muscle of each pig in groups 1, 2, and 3. The piglets in group 4 were inoculated with the same volume of PBS as the mock group. The clinical signs (scores) and body temperatures of the piglets were measured every day for 21 days, and the morbidity and mortality in each group were recorded. Nasal swabs, anal swabs and serum samples were collected at 1, 3, 5, 7, 10, 14, and 21 dpc. All pigs were euthanized with sodium pentobarbital (25 mg/kg) at 21 dpc.

### Detection of antibodies against PRRSV

Serum samples were collected at 0, 1, 3, 5, 7, 10, 14, and 21 dpc (safety evaluation), and 0, 7, 14, and 21 dpv and 3, 5, 7, 14, and 21 dpc (immunoprotection evaluation) were used to specifically test for anti-PRRSV-2 antibodies via nanobody-based cELISA as previously described [23]. Additionally, a commercial ELISA kit (IDEXX Laboratories, Westbrook, ME, USA) was used to test anti-PRRSV-1 and -2 common antibodies in the serum samples according to the manufacturer’s instructions. The serum samples were considered positive if the S/P ratio was 0.4 or higher.

### Detection of viremia and viral loads in the collected samples

Total RNA was extracted from blood and nasal and anal swab samples collected from the pigs according to the TRIzol protocol (TaKaRa, Tokyo, Japan). The copy numbers of PRRSV RNA were detected via RT‒PCR with a fluorescent TaqMan according to the manufacturer’s instructions (Wuhan Grint Biology).

### Pathological and immunohistochemical analyses

At necropsy, the lungs were collected to observe gross lesions and immersed in formalin for histopathological examination. Gross lung lesions were recorded and scored by a pathologist [31]. The hematoxylin and eosin (H&E) protocol was used to visualize pathological changes in the lung samples as previously described [32]. The results were scored by the same pathologist [31].

### Statistical analysis

Significant differences between the 4 groups were analysed with GraphPad Prism 6.01 (GraphPad Software, California, USA). All the data are presented as the means ± SEM unless otherwise stated, and the asterisks and pound signs in the figures indicate statistical significance (*P < 0.05, **P < 0.01, ***P < 0.001, and ****P < 0.0001).

## Results

### Rescue of recombinant rSX-HD and chimeric rSX-HD^2M1^

After sequencing, the complete genome of PRRSV-2 SX-HD was successfully inserted into the pBAC vector (Figure 1A). Another chimeric infectious clone was named pBAC-rSX-HD^2M1^, in which the genes encoding the N protein from PRRSV-1 GZ11-G1 were successfully used to replace the genes encoding the N protein in pBAC-rSX-HD (Figure 1B). The positive plasmids pBAC-rSX-HD and pBAC-rSX-HD^2M1^ were subsequently transfected into Marc-145 cells, and the cell density reached 80%. At 72 hpi, CPEs in the two transfected Marc-145 cell lines were observed, and the cells exhibited shrinkage and clumping (Figure 1C). The rescued viruses were harvested by three freeze‒thaw cycles and separately named rSX-HD and rSX-HD^2M1^. Additionally, the IFA results revealed green fluorescence in Marc-145 cells infected with wild-type SX-HD or rSX-HD by primary antibodies against 6D10 and PRRSV-N-Nb1 (Figure 1D). However, green fluorescence was detected only in Marc-145 cells infected with rSX-HD when stained with the primary antibody PRRSV-N-Nb1, whereas no signal was detected in Marc-145 cells infected with rSX-HD^2M1^ (Figure 1E). The rSX-HD and rSX-HD^2M1^ stocks were collected and used to extract the viral RNA. The viral RNA was subsequently sequenced, and the results revealed 100% identity with the sequences in pBAC-SX-HD and pBAC-SX-HD^2M1^ (data not shown). These results suggested that rSX-HD and rSX-HD^2M1^ were successfully rescued. The rescued rSX-HD^2M1^ virus was attenuated by passaging Marc-145 cells for 120 passages over a period of approximately one year.

After the growth kinetics of the parental virus (SX-HD) and the different generations of rSX-HD^2M1^ (F1, F10, F20, F30········F120) were determined, the results revealed that they were significant (*P* < 0.05) (Figure 1F). However, the first-generation (F1) titre was 10^5.4^ TCID_50_/mL (Figure 1F). With increasing passages, the titres of the progeny virus also increased. The virus proliferation ability reached the highest value (10^7.7^ TCID_50_/mL) at approximately the F100 generation and tended to be stable at the F120 generation (Figure 1F).

### Amino acid mutations of rSX-HD^2M1^ in each of the 10 generations

Viral RNA was extracted from the different generations (F1, F10, F20, F30········F120) and sequenced via next-generation sequencing (NGS). The results revealed that seventeen amino acids were mutated from the first passage to the 120th passage (Table 2). The major changes were located in Nsp2 and minor structural proteins GP2, GP4, GP5 and M. There were 7 amino acid mutations in the Nsp2 proteins, including Asn308Asp, Lys397Glu, Ser446Arg, Glu550Gly, Glu554Lys, Ser763Leu, and Tyr806Phe, in which 2 amino acids were mutated in the first 10 generations, and most amino acid mutations were concentrated from generations 50–60. Additionally, because the GP5 of PRRSV induces mainly neutralizing antibodies, we also analysed the mutations of the protein, and two amino acid mutations were detected in the protein, namely, Asn34Asp and Gln196Arg (Table 2). These genes were mutated in the first 10 generations (Table 2). Additionally, the chimeric PRRSV-1 N gene was very stable from the first passage to the 120th passage, and no nucleotide or amino acid mutations or deletions were observed.

**Table 2:** **Mutations of different proteins across passages**

Positions of different protein mutations		Different passage of rSX-HD^2M1^												
		F1	F10	F20	F30	F40	F50	F60	F70	F80	F90	F100	F110	F120
Nsp2	308	N	D	D	D	D	D	D	D	D	D	D	D	D
	397	K	K	E	E	E	E	E	E	E	E	E	E	E
	446	S	S	S	S	S	S	R	R	R	R	R	R	R
	550	E	E	E	E	E	E	E	G	G	G	G	G	G
	554	E	E	E	E	E	E	E	K	K	K	K	K	K
	763	S	L	L	L	L	L	L	L	L	L	L	L	L
	806	Y	Y	Y	Y	Y	F	F	F	F	F	F	F	F
	50	Y	Y	Y	Y	Y	Y	F	F	F	F	F	F	F
GP2	118	I	I	T	T	V	V	V	V	V	V	V	V	V
	132	S	S	S	S	N	N	N	N	N	N	N	N	N
GP4	150	H	H	H	H	R	R	R	R	R	R	R	R	R
	172	F	F	F	F	F	F	F	F	V	V	V	V	V
GP5	34	N	D	D	D	D	D	D	D	D	D	D	D	D
	196	Q	R	R	R	R	R	R	R	R	R	R	R	R
M	160	K	R	R	R	R	R	R	R	R	R	R	R	R
	173	A	G	G	G	G	G	G	G	G	G	G	G	G
	174	K	R	R	R	R	R	R	R	R	R	R	R	R

### Safety of rSX-HD^2M1^-F120 for piglets

**Figure 2:**
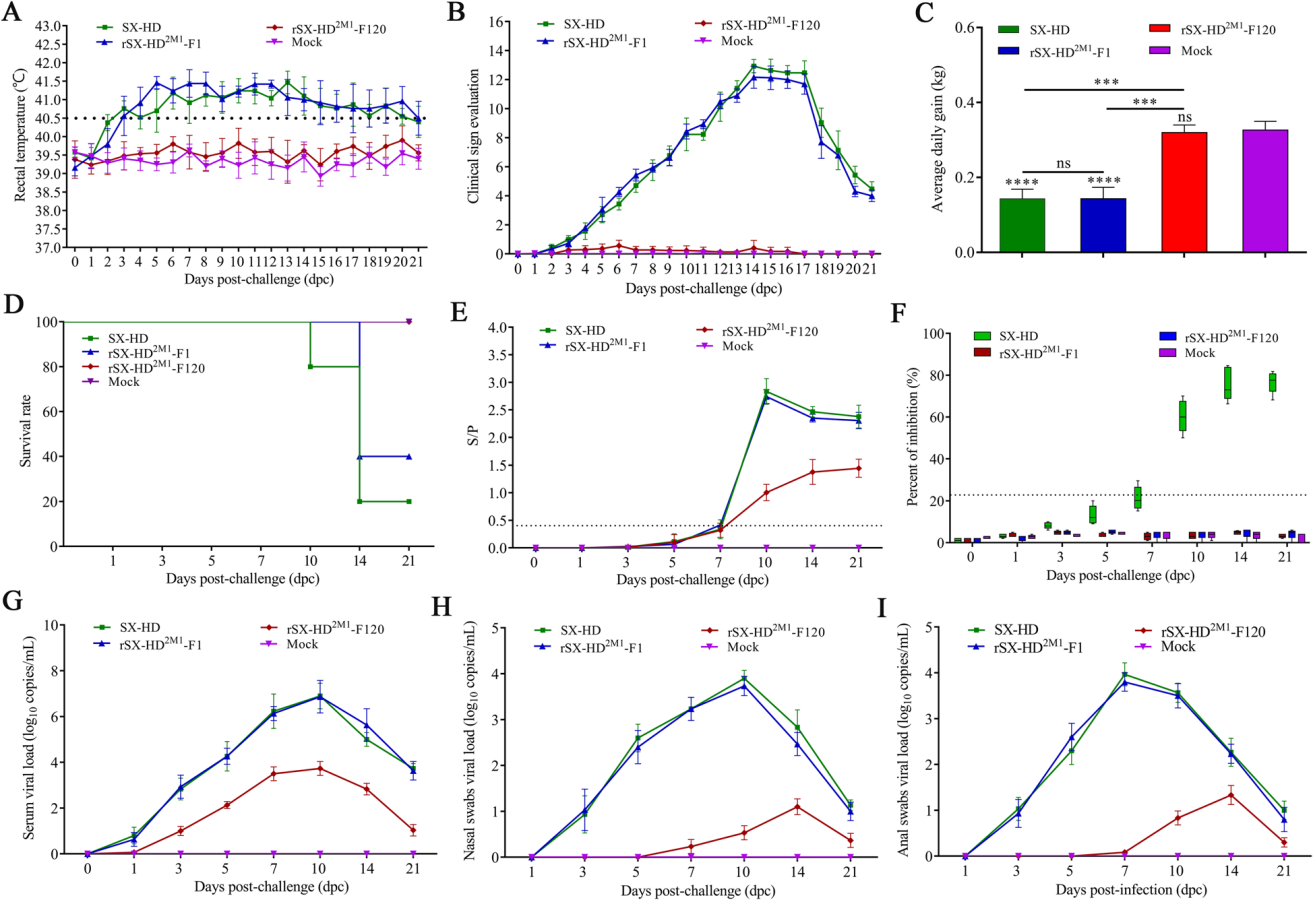
**Safety evaluation of rSX-HD**^**2M1**^**-F120 in piglets**. **A** Body temperature changes of the piglets in the SX-HD, rSX-HD^2M1^-F1, rSX-HD^2M1^-F120 and mock groups. **B** Clinical scores of the piglets in the SX-HD, rSX-HD^2M1^-F1, rSX-HD^2M1^-F120 and mock groups. **C** Body weight changes in the piglets in the SX-HD, rSX-HD^2M1^-F1, rSX-HD^2M1^-F120 and mock groups. **D** Survival rates of piglets in the SX-HD, rSX-HD^2M1^-F1, rSX-HD^2M1^-F120 and mock groups. **E** Detection of anti-PRRSV antibodies in piglets from the SX-HD, rSX-HD^2M1^-F1, rSX-HD^2M1^-F120 and mock groups during the experimental study via a commercial IDEXX ELISA kit. **F** Detection of anti-PRRSV-2 antibodies in piglets from the SX-HD, rSX-HD^2M1^-F1, rSX-HD^2M1^-F120 and mock groups during the experimental study via nanobody-based cELISA. **G** Detection of viral loads in the blood of piglets from the SX-HD, rSX-HD^2M1^-F1, rSX-HD^2M1^-F120 and mock groups. **H** Detection of viral loads in nasal secretions from piglets in the SX-HD, rSX-HD^2M1^-F1, rSX-HD^2M1^-F120 and mock groups. **I** Detection of viral loads in the anal secretions of piglets from the SX-HD, rSX-HD^2M1^-F1, rSX-HD^2M1^-F120 and mock groups. Five pigs were included in each group. Significant differences are marked with asterisks and “ns”: ****P < 0.0001; ***P < 0.001; ** *P* < 0.01.

Compared with those in the SX-HD group, piglets immunized with rSX-HD^2M1^-F120 did not present any overt clinical signs of PRRSV infection, including changes in behavior, body temperature or body weight (Figures 2A‒C). After infection, the piglets in the SX-HD and rSX-HD^2M1^-F1 groups presented multiple disease manifestations starting as early as 2 dpc, including anorexia, skin cyanopathy, chemosis, diarrhea, coughing or claudication, whereas those in the rSX-HD^2M1^-F120 and mock groups presented no clinical signs. The body temperatures of pigs in the rSX-HD^2M1^-F120 and mock groups remained normal throughout the experiment, but the pigs in the SX-HD and rSX-HD^2M1^-F1 groups presented persistently high fevers, with increased mean rectal temperatures above 40.5 °C (Figure 2A). An evaluation of the scores of clinical signs revealed that the pigs in the SX-HD and rSX-HD^2M1^-F1 groups presented higher scores for clinical signs than those in the rSX-HD^2M1^-F120 and mock groups did (Figure 2B). The average daily weight gain (ADWG) in each group was calculated. The mean weights of the piglets in the rSX-HD^2M1^-F120 and mock groups were significantly greater than those in the rSX-HD and rSX-HD^2M1^-F1 groups. There were no significant differences between the rSX-HD^2M1^-F120 and mock groups during the experimental period (Figure 2C).

In addition, the piglets in the rSX-HD and rSX-HD^2M1^-F1 groups died between 12 and 21 dpc, and the mortality rates reached 80 and 60%, respectively. All the pigs inoculated with rSX-HD^2M1^-F120 survived during the experimental period (Figure 2D).

The detection of anti-PRRSV antibodies with commercial IDEXX ELISA kits revealed that seroconversion occurred at 7 dpc in the SX-HD and rSX-HD^2M1^-F1 groups. The remaining piglets were all positive for anti-PRRSV antibodies at 10 dpc (Figure 2E). Three piglets in the rSX-HD^2M1^-F120 group were seroconverted at 7 dpc. The remaining piglets also seroconverted at 10 dpc (Figure 2E). No specific anti-PRRSV antibodies were detected in the piglets in the mock group throughout the experiment (Figure 2E).

The detection of anti-PRRSV-2-N antibodies via nanobody-based cELISA revealed that no specific anti-PRRSV-2-N antibodies were detected during the experimental period in the piglets of the rSX-HD^2M1^-F1, rSX-HD^2M1^-F120 and mock groups (Figure 2F). However, the piglets in the SX-HD group were seroconverted at 7 dpc according to cELISA (Figure 2F).

To evaluate viremia and the duration of PRRSV infection, serum, nasal and anal swab samples collected at 0, 1, 3, 7, 10, 14, and 21 dpc were evaluated via RT‒PCR. In both the SX-HD and the rSX-HD^2M1^-F1 groups, PRRSV RNA was detected in the serum samples at 1 dpc, with peak viral titres at 10 dpc. Following the peak, the viral titres decreased slightly in the serum. In the rSX-HD^2M1^-F120 group, PRRSV RNA was detected in the serum at 3 dpc, and the viral titres were much lower than those in the two groups described above. No viremia was detected in the control groups at any point in time (Figure 2G). PRRSV RNA was detected in nasal and anal swabs at 3 dpc, with peak viral shedding at 7 and 10 dpc, respectively. Respiratory viral shedding and gastrointestinal shedding were significantly greater in SX-HD- and rSX-HD^2M1^-F1-infected pigs than in rSX-HD^2M1^-F120-infected pigs during the experimental period (Figures 2H, I).

**Figure 3:**
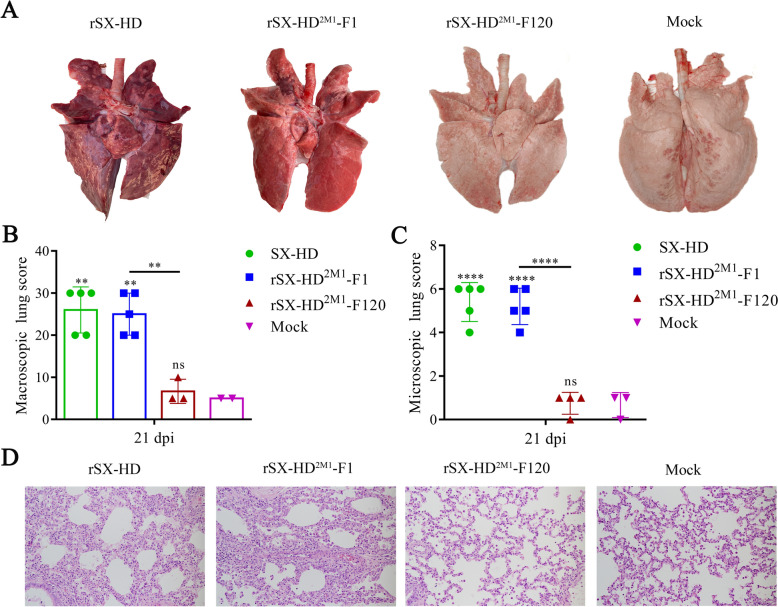
**Gross and microscopic lesions in piglet lungs from the SX-HD, rSX-HD**^**2M1**^**-F1, rSX-HD**^**2M1**^**-F120 and mock groups**. **A** Gross lesions in piglet lungs. Piglets from the SX-HD- and rSX-HD^2M1^-F1-challenged groups presented severe interstitial pneumonia. Piglets from the rSX-HD^2M1^-F120-challenged and mock groups presented no gross lesions. The solid arrows indicate interstitial pneumonia and pulmonary congestion. **B** Scores of piglet lungs were calculated by assigning a numerical value to each lobe, representing the approximate percentage of the entire lung represented by that lobe. **C** The assessment of the degree and severity of interstitial pneumonia was considered in the evaluation of microscopic lesions and the scoring of lung tissues: 0, no lesion; 1, mild/focal; 2, moderate/multifocal; 3, moderate/diffuse; and 4, severe/diffuse. Three pathologists evaluated the lung lesions, both microscopically and macroscopically, via blind examinations. **D** Microscopy images of lesions in piglet lungs. Piglets from the SX-HD- and rSX-HD^2M1^-F1-challenged groups presented thickened alveolar septa, alveolar epithelial cell degeneration, and inflammation. Piglets from the rSX-HD^2M1^-F120-challenged and mock groups had no evident lung pathology. The solid arrows indicate thickening of the interlobular septa or infiltration of inflammatory cells around the bronchioles. Magnification 100×. Significant differences are marked with asterisks and “ns”: **P < 0.01, ****P < 0.0001.

The piglets in the SX-HD- and rSX-HD^2M^-F1-infected groups presented typical gross lesions of PRRS, including consolidation, firmer and dense parenchyma in the lung tissues with hemorrhage (Figure 3A). No obvious gross lesions were observed in the rSX-HD^2M1^-F120-infected and control groups (Figures 3A, B). Histologic analysis revealed a large amount of inflammatory cell infiltration and significant widening of the alveolar diaphragm in the lungs of the piglets in the SX-HD- and rSX-HD^2M^-F1-infected groups. No microscopic lesions were observed in the rSX-HD^2M1^-F120-infected or control groups (Figures 3C, D). These data indicated that rSX-HD^2M1^-F120 was safe for piglets.

### Clinical evaluation after immunization and challenge

**Figure 4:**
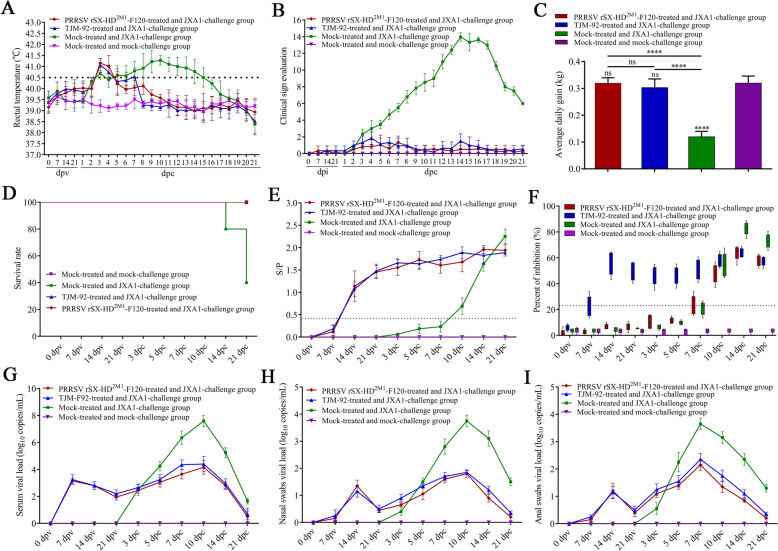
**Protection analysis of piglets immunized with the PRRSV rSX-HD**^**2M1**^**-F120 strain**. **A** Body temperature changes in piglets in the PRRSV rSX-HD^2M1^-F120-treated and JXA1-challenged, TJM-92-treated and JXA1-challenged, mock-treated and JXA1-challenged, and mock-treated and mock-challenged groups. **B** Clinical scores of piglets in the PRRSV rSX-HD^2M1^-F120-treated and JXA1-challenged, TJM-92-treated and JXA1-challenged, mock-treated and JXA1-challenged, and mock-treated and mock-challenged groups throughout the entire experiment. **C** Body weight changes in piglets in the PRRSV rSX-HD^2M1^-F120-treated and JXA1-challenged, TJM-92-treated and JXA1-challenged, mock-treated and JXA1-challenged, and mock-treated and mock-challenged groups during the experiment. **D** Survival rates of piglets in the PRRSV rSX-HD^2M1^-F120-treated and JXA1-challenged, TJM-92-treated and JXA1-challenged, mock-treated and JXA1-challenged, and mock-treated and mock-challenged groups. **E** PRRSV-specific antibody levels were detected by a commercial ELISA kit in piglets from the PRRSV rSX-HD^2M1^-F120-treated and JXA1-challenged, TJM-92-treated and JXA1-challenged, mock-treated and JXA1-challenged, and mock-treated and mock-challenged groups during the challenge study. **F** PRRSV-specific antibody levels were detected by nanobody-based cELISA in piglets from the PRRSV rSX-HD^2M1^-F120-treated and JXA1-challenged groups, TJM-92-treated and JXA1-challenged groups, mock-treated and JXA1-challenged groups, and mock-treated and mock-challenged groups. **G** Viral load detection in the blood of all the piglets. **H** Detection of viral loads in nasal secretions from all the piglets. **I** Detection of viral loads in anal secretions from all the piglets. Significant differences are marked with asterisks and “ns”: ****P < 0.0001.

During the experimental period, no clinical signs of PRRS, including changes in behavior, body temperature or body weight, were observed in the piglets in the rSX-HD^2M1^-F120-treated or commercial attenuated vaccine immunization (TJM-F92-treated) groups following challenge with JXA1 or mock-treated and mock-challenged groups (Figures 4A, B). However, the piglets in the mock-treated and JXA1-challenged groups presented various disease manifestations, including persistent fever from 7–14 dpc (≥40.5 °C) (Figure 4A), anorexia, skin cyanopathy, chemosis, coughing, shivering or lameness (Figure 4B). In particular, with respect to body weight, all the piglets in the rSX-HD^2M1^-F120-treated and TJM-F92-treated groups following challenge with JXA1 and the mock-treated and mock-challenged groups gained weight at a greater rate (*P* < 0.0001) than did those in the mock-treated and JXA1-challenged groups (Figure 4C). The mortality rate of the mock-treated and JXA1-challenged groups was 60%. Three piglets died at 14, 17 and 21 dpc. All the pigs in the other groups survived throughout the entire experimental period (Figure 4D).

### Antibody responses in immunized piglets

The detection of anti-PRRSV antibodies via commercial ELISA revealed that all immunized piglets in the rSX-HD^2M1^-F120-treated and TJM-F92-treated groups were seroconverted at 14 dpv (Figure 4E). After challenge, all the piglets in the mock-treated and JXA1-challenged groups also exhibited seroconversion at 10 dpc (Figure 4E). No PRRSV-specific antibodies were detected in the control piglets prior to challenge (Figure 4E). The antibody responses of piglets in the mock group were negative throughout the experiment (Figure 4E).

The anti-PRRSV-2 antibodies detected via cELISA revealed that no anti-PRRSV-2 antibodies were detected during the 21-day immunization period in the piglets of the rSX-HD^2M1^-F120-treated and mock-treated groups (Figure 4F). The piglets in the TJM-F92-treated groups were seroconverted at 7 dpc (Figure 4F). However, after challenge, the piglets in the rSX-HD^2M1^-F120-treated groups challenged with JXA1 and those in only the JXA1-challenged groups were seroconverted at 7 dpc (Figure 4F). The antibody responses of the piglets in the mock-treated and mock-challenged groups were all negative throughout the experiment (Figure 4F).

### Virus detection in challenged piglets

To further evaluate viremia in the piglets of different groups, serum samples from 0, 7, 14, and 21 dpv and 1, 3, 5, 7, 10, 14, and 21 dpc were collected and tested by RT‒qPCR. The results revealed that the PRRSV RNA copies in the serum samples from the rSX-HD^2M1^-F120-treated and TJM-92-treated groups reached their highest levels at 7 dpv and then gradually declined (Figure 4G). At 21 dpc, PRRSV RNA was no longer detectable in the piglets of the rSX-HD^2M1^-F120-treated and TJM-92-treated groups (Figure 4G). Additionally, from 5 to 21 dpc, the results revealed that the number of PRRSV RNA copies in the serum of piglets in the rSX-HD^2M1^-F120-treated and TJM-92-treated groups was significantly lower than that in the serum of piglets in the only JXA1-challenged group (Figure 4G).

For viral shedding, PRRSV RNA was detected in the nasal and anal swabs of the rSX-HD^2M1^-F120-treated and TJM-92-treated groups at 7 dpv and peaked at 14 dpv (Figures 4H, I). After challenge, PRRSV RNA was detected only in the JXA1-challenged group at 3 dpc and peaked at 10 dpc (Figure 4H, I). Additionally, respiratory and gastrointestinal viral shedding was significantly greater in the mock-treated and JXA1-challenged groups than in the rSX-HD^2M1^-F120-treated and TJM-92-treated groups at 7 and 21 dpc (Figures 4H, I). These results indicate that the number of PRRSV RNA copies in the serum and nasal and anal swabs of challenged piglets can be significantly reduced after they are immunized with TJM-F92 or rSX-HD^2M1^-F120.

### Pathological and histopathological examination

**Figure 5:**
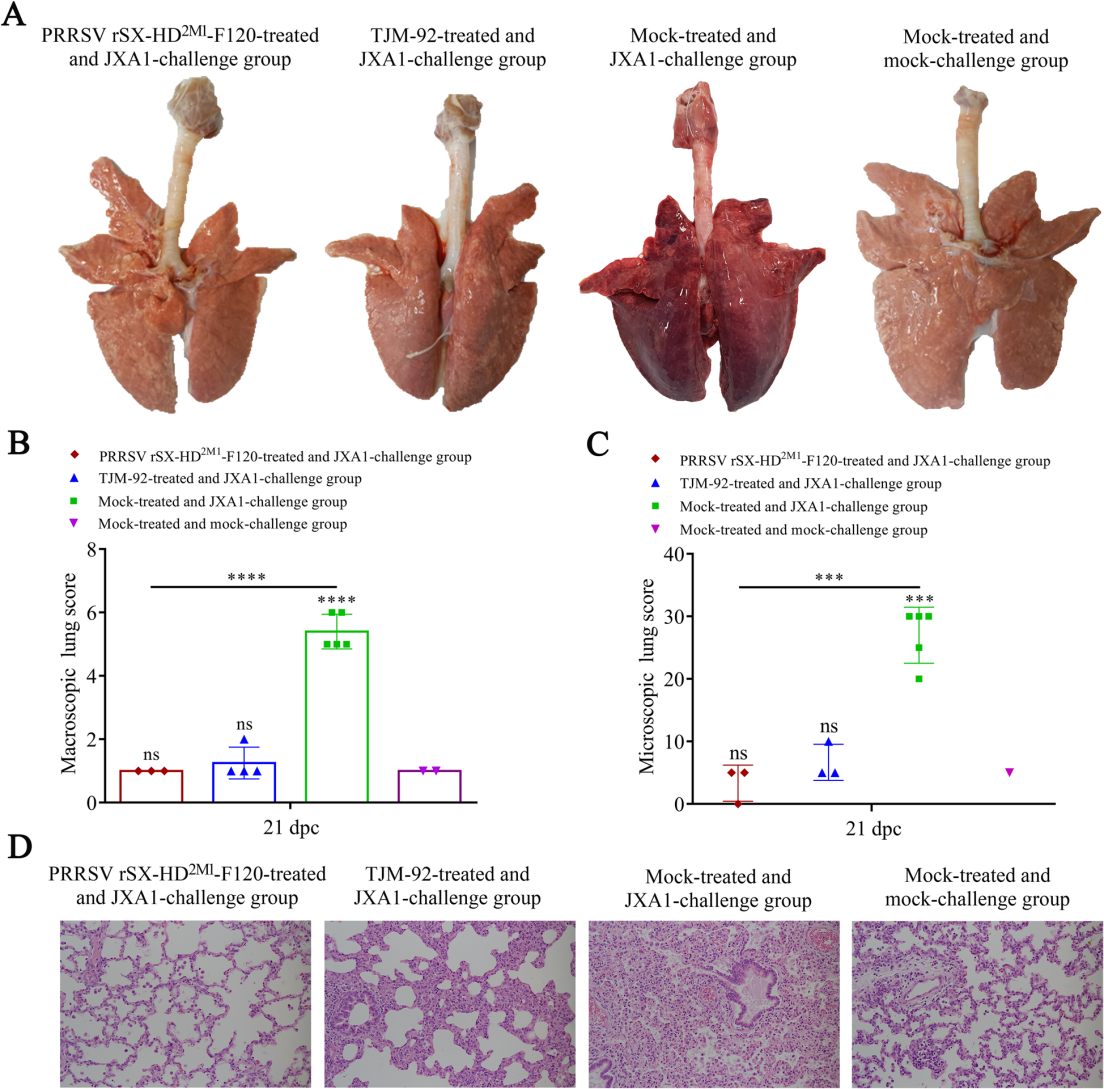
**Gross and microscopic lesions in piglet lungs**. **A** Gross lesions in piglet lungs. Piglets from the JXA1 challenge group developed severe interstitial pneumonia. Piglets from the PRRSV rSX-HD^2M1^-F120-treated and JXA1-challenged, TJM-92-treated and JXA1-challenged, and mock-treated and mock-challenged groups presented no gross lung lesions. The solid arrows indicate interstitial pneumonia and pulmonary congestion. **B** Scores of piglet lungs were calculated by assigning a numerical value to each lobe, representing the approximate percentage of the entire lung represented by that lobe. **C** The assessment of the degree and severity of interstitial pneumonia was considered in the evaluation of microscopic lesions and the scoring of lung tissues: 0, no lesion; 1, mild/focal; 2, moderate/multifocal; 3, moderate/diffuse; and 4, severe/diffuse. Three pathologists evaluated the lung lesions, both microscopically and macroscopically, via blind examinations. **D** Microscopy images of lesions in piglet lungs. Piglets from the JXA1 challenge group presented thickened alveolar septa, alveolar epithelial cell degeneration, and inflammation. Piglets in the PRRSV rSX-HD^2M1^-F120-treated and JXA1-challenged, TJM-92-treated and JXA1-challenged and mock-treated and mock-challenged groups presented no evident lung pathology. The solid arrows indicate thickening of the interlobular septa or infiltration of inflammatory cells around the bronchioles. Magnification 100×. Significant differences are marked with asterisks and “ns”: ***P < 0.001, ****P < 0.0001.

Compared with the piglets in the rSX-HD^2M1^-F120-treated, TJM-92-treated and mock groups, the piglets in the mock-treated and JXA1-challenged groups presented typical gross lesions of PRRS, including consolidation, firmer and dense parenchyma in the lung tissues with hemorrhage (Figures 5A, B). No obvious gross lesions were observed in the other three groups (Figures 5A, B). Histologic analysis revealed a large amount of inflammatory cell infiltration and significant widening of the alveolar diaphragm in the lungs of the piglets in the mock-treated and JXA1-challenged groups (Figures 5C, D). Notably, the pathological damage to the lungs of the piglets in the TJM-F92-treated groups was mild compared with that in the mock-treated and JXA1-challenged groups, and no microscopic lesions were observed in the rSX-HD^2M1^-F120-treated group (Figures 5C, D). These data indicate that rSX-HD^2M1^-F120 can reduce the number of gross and microscopic lesions in the lungs of vaccinated pigs following challenge with the HP-PRRSV JXA1 strain.

## Discussion

The eradication of animal diseases is an important measure for disease prevention and control [33]. An increasing number of countries have implemented PRRSV eradication programs that focus on the detection and removal of persistently infected pigs [34]. However, owing to the lack of efficient and easy-to-operate serological markers or DIVA vaccines, the prevention and control of PRRSV infection were carried out mainly through MLV immunization [12]. In this study, a serologic marker attenuated live vaccine candidate strain based on HP-PRRSV was developed for the first time. Combined with our developed nanobody-based cELISA, this vaccine candidate can be used for the eradication of PRRSV-2 infection from pig farms in the future.

The most important step in the development of a DIVA vaccine is the identification of a potential serological marker antigen [35]. Since a DIVA vaccine is only useful with a companion diagnostic test, the marker antigen should be conserved and immunodominant to ensure that the companion diagnostic test based on this marker antigen will yield reliable differential detection [12]. Previously, several DIVA vaccines for PRRSV based on Nsp2 were developed [36, 37]. However, owing to the high genetic variation of the different PRRSV Nsp2 genes and the early detection of antibodies against PRRSV Nsp2 in pigs at 14 dpc, these DIVA vaccines have not been commercialized [38]. The PRRSV N gene is relatively conserved and has good immunogenicity [39]. Importantly, antibodies against the PRRSV N protein can be detected early, at 7 days after PRRSV infection [16, 39]. On the basis of the biological and immunological functions of the PRRSV N protein, in the present study, we first proposed the use of N protein substitution as a serological marker for the development of a live attenuated DIVA vaccine against HP-PRRSV.

Current attenuated PRRSV vaccines are produced by serial passaging of Marc-145 cells in vitro. To adapt to the proliferation of PRRSV in cells, some adaptive mutations occur during the process of passaging. For example, one study identified two amino acids that are mutated in the GP5 protein: Q^196^ → R ^196^ and N^34^ → D^34^ [27]. Another study revealed that 4 amino acids (F^23^ → S^23^, G^80^ → V^80^, R^151^ → K^151^, and Q^196^ → R^196^) are mutated in the GP5 protein [40]. In the present study, rSX-HD^2M1^-F120, a genetically stable attenuated viral strain, was also obtained by serial passaging of Marc-145 cells. For the different generations (F1–F120), sequence alignment revealed that the major changes in different PRRSV proteins were located on Nsp2 and the minor structural proteins GP2, GP4, GP5 and M. All amino acid mutations related to SX-HD were observed before the 60th passage. We also found two mutation sites on GP5 (Q^196^ → R^196^ and N^34^ → D^34^), which is the same as HUN4/HUN4-F112 [27]. No nucleotide or amino acid mutations were observed in the N protein. This finding indicated that the rSX-HD strain at the 60th passage had adapted to Marc-145 cells and was subsequently stably passaged.

Commercial attenuated live HP-PRRSV vaccines, including JXA1-R, TJM, HuN4-F112, and GDr180, were obtained through the passage of the respective PRRSV strains in Marc-145 cells for 80, 92, 112, and 180 passages, respectively [27, 41]. In the present study, rSX-HD^2M1^ expression was attenuated by passage for 120 generations in Marc-145 cells. Animal experiments also revealed that rSX-HD^2M1^-F120 inoculation was safe for piglets, as it resulted in no fever or clinical symptoms. However, the inoculated piglets also showed viremia, which was the same as that of other attenuated live vaccine strain inoculations. The duration of viremia varied among the different attenuated live PRRSV-2 vaccines with different immune doses. For example, after piglets were immunized with HP-PRRS vaccines at a dose of 10^5.0^ TCID_50_, the duration of viremia in some pigs was approximately 3 to 21 days (JXA1-P80: 3~14 days; HuN4-F112: 7~14 days; TJM-F92: 3~21 days) [27, 40, 42]. In the present study, the viremia duration of the piglets inoculated with rSX-HD^2M1^-F120 was also 3–21 days. In the future, novel strategies should be designed for clearing or shortening viremia after attenuated live PRRSV vaccines are used to immunize piglets.

Frequent recombination of PRRSV strains is an important evolutionary mechanism that profoundly influences viral replication dynamics and virulence modulation [43]. This genetic adaptability poses substantial challenges to the prevention and control of PRRSV [44]. In our study, the divergent lethality observed between piglets inoculated with the SX-HD strain (safety experiment) and those inoculated with the JXA1 strain (protection experiment) highlights substantial variations in virulence among different HP-PRRSV isolates. These differences likely stem from evolutionary mechanisms driving virulence adaptation in PRRSV populations. Numerous studies in China have reported recombination between HP-PRRSV and NADC30-like strains [45, 46]. Notably, such recombination has been implicated as a key driver of the widespread dissemination of NADC30-like variants since their initial emergence in 2013 [47]. These findings raise biosafety concerns regarding potential recombination events between the attenuated vaccine strain rSX-HD^2M1^-F120 and circulating wild-type strains (particularly JXA1-like or NADC30-like lineages). To address this risk, future vaccine development should prioritize replication-defective candidates derived from the rSX-HD^2M1^-F120 platform. Such next-generation vaccines would theoretically maintain protective immunogenicity while eliminating recombination risks associated with replicating viral components.

In this study, an attenuated live PRRSV-2 vaccine candidate was designed and developed on the basis of the genome sequence of the HP-PRRSV strain. However, the prevalence of PRRSV NADC30-like and NADC34-like strain infections has recently increased on pig farms in China [7, 8]. Whether this serologic marker PRRSV-2 vaccine can offer cross-protection against NADC30-like and NADC34-like strain infections in pigs may require additional animal experiments for verification. Nevertheless, the platform for the development of a serologic marker vaccine was designed on the basis of the highly conserved PRRSV-2 N protein. The developed nanobody-based cELISA can detect antibodies against all PRRSV-2 isolates. Thus, in the future, recombinant chimeric infectious clones can also be constructed on the basis of the genome sequences of epidemic PRRSV-2 strains, after which the gene encoding the PRRSV-2 protein can be replaced with that encoding the PRRSV-1 N protein. For example, the NADC30-like strain can be used as a backbone to construct chimeric PRRSV, which is subsequently attenuated in Marc-145 cells as a serologic marker vaccine candidate against NADC30-like strain infection in pigs.

In summary, for the first time, we have presented a strategy to replace the serologically marked epitope of the N protein for developing a DIAV PRRSV-2 vaccine candidate. Animal experiments revealed that rSX-HD^2M1^-F120 was safe for piglets and could provide protection against HP-PRRSV JXA1 challenge. Importantly, rSX-HD^2M1^-F120 immunization combined with the developed nanobody-based cELISA can differentiate attenuated live vaccine candidate immunization from wild-type PRRSV-2 infection in pigs. Therefore, the DIVA vaccine based on the candidate rSX-HD^2M1^-F120 provides a foundation for the eradication of PRRSV-2 infection in pig farms in China.

## Data Availability

The datasets used or analysed during the current study are available from the corresponding author upon reasonable request.
